# Two novel mutations in TCIRG1 induced infantile malignant osteopetrosis: a case report

**DOI:** 10.1186/s12887-021-02774-1

**Published:** 2021-07-01

**Authors:** Ping Wu, Zhe Cai, Wen-Hui Jiang, Gen Lu, Pei-Qiong Wu, Zhi-Wei Xie, Jun-Zheng Peng, Chen Chen, Jun-Ye Qi, Li-Zhen Xu, Kun-Ling Shen, Hua-Song Zeng, Gen-Quan Yin

**Affiliations:** 1 Department of Respirology, Guangzhou Women and Children’s Medical Center, Guangzhou Medical University, Guangzhou, 510120 Guangdong China; 2 Department of Respirology, Beijing Children’s Hospital, Capital Medical University, Beijing, 100045 China; 3 Department of Allergy, Immunology and Rheumatology, Guangzhou Women and Children’s Medical Center, Guangzhou Medical University, Guangzhou, 510120 Guangdong China; 4 Guangzhou Institute of Pediatrics, Guangzhou Women and Children’s Medical Center, Guangzhou, 510623 China; 5 Institute Pasteur of Shanghai, Chinese Academy of Science, Shanghai, 200031 China; 6 Department of Chemical Pathology, The Chinese University of Hong Kong, Prince of Wales Hospital, Hong Kong, China

**Keywords:** Infantile malignant osteopetrosis, TCIRG1, Mutation, Autosomal recessive osteopetrosis

## Abstract

**Background:**

Infantile malignant osteopetrosis (IMO) is a rare autosomal recessive disease characterized by a higher bone density in bone marrow caused by the dysfunction of bone resorption. Clinically, IMO can be diagnosed with medical examination, bone mineral density test and whole genome sequencing.

**Case presentation:**

We present the case of a 4-month-old male infant with abnormal skull development, hypocalcemia and premature closure of the cranial sutures. Due to the hyper bone density showed by his radiographic examination, which are characteristic patterns of IMO, we speculated that he might be an IMO patient. In order to confirm this diagnosis, a high-precision whole exome sequencing of the infant and his parents was performed. The analysis of high-precision whole exome sequencing results lead to the identification of two novel heterozygous mutations c.504-1G > C (a splicing site mutation) and c.1371delC (p.G458Afs*70, a frameshift mutation) in gene TCIRG1 derived from his parents. Therefore, we propose that there is a close association between these two mutations and the onset of IMO.

**Conclusions:**

To date, these two novel mutations in gene TCIRG1 have not been reported in the reference gene database of Chinese population. These variants have likewise not been reported outside of China in the Genome Aggregation Database (gnomAD). Our case suggests that the use of whole exome sequencing to detect these two mutations will improve the identification and early diagnosis of IMO, and more specifically, the identification of homozygous individuals with TCIRG1 gene mutation. We propose that these mutations in gene TCIRG1 could be a novel therapeutic target for the IMO in the future.

## Background

Osteopetrosis, a rare group of genetic skeletal diseases, is characterized by an increased bone density of entire skeletal system. The overall incidence of osteopetrosis is about 1/20,000 [1, 2]. Osteopetrosis includes two genetic patterns, one of which is autosomal dominant inheritance that is usually asymptomatic or is inadvertently diagnosed in late childhood. The other is autosomal recessive inheritance. IMO belongs to the autosomal recessive osteopetrosis (ARO), which is usually diagnosed after birth or during the fetal period. IMO patients may develop severe symptoms, including bone marrow failure, hepatosplenomegaly, and macrocephaly with the early closing of the fontanel. More than 70% of IMO cases are induced by changes associated with pathogenic genes [3]. There are at least 10 pathogenic genes that have been identified in humans [4]. IMO is one of the most severe types of ARO, which is often fatal within the first several years of life. Hence, the early transplantation of hematopoietic stem cell remains the only curative treatment for ARO [5]. More than 50% of IMO cases are attributed to the T-cell immune regulator 1 (TCIRG1) gene mutation [4]. This gene mutation caused the autosomal recessive disease known as type 1 osteopetrosis. Some of the most common clinical manifestations of IMO include systemic osteosclerosis, excessive growth of cranial nerve and magnum foramen caused cranial nerve compression [6–10]. However, early diagnosis of susceptible gene mutation and the subsequent early treatment of osteopetrosis are lacking. As such, clinicians are actively researching on the early diagnosis of susceptible gene mutation of osteopetrosis.

Here, we report a case featuring an IMO pediatric patient who has two novel mutations in gene TCIRG1. These gene mutations are c.504-1G > C and c.1371delC (p.G458Afs*70), which are inherited from the heterozygous parents of this child. So far, these two novel mutations have not been reported in and outside of Chinese population in the reference gene database (Genome Aggregation Database) (https://gnomad.broadinstitute.org/gene/ENSG00000110719?dataset=gnomad_r2_1). We propose that these mutations in gene TCIRG1 are potential clinical markers for the early diagnostics of IMO.

## Case presentation

### The discovery of TCIRG1 mutation

**Fig. 1:**
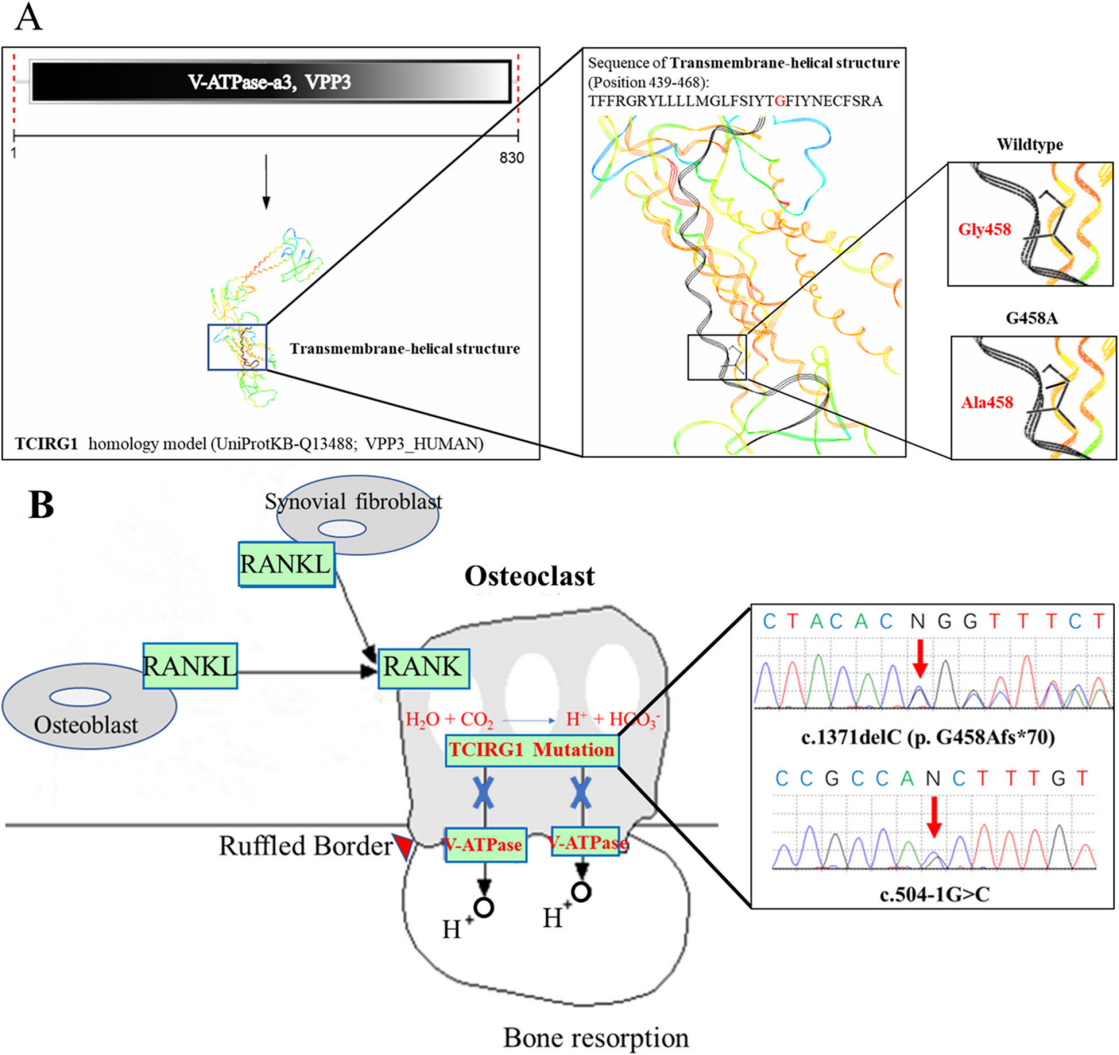
The pathogenesis mechanism of infantile malignant osteopetrosis. **A**. The mutation site in TCIRG1 homologous model. The overall protein VPP3 structure is shown in rainbow color. The transmembrane-helical structure is shown in black flat line. The specific residue and substitution are shown as sticks. **B**. The assumed intracellular process of infantile malignant osteopetrosis via TCIRG1 mutation in osteoclast

**Fig. 2:**
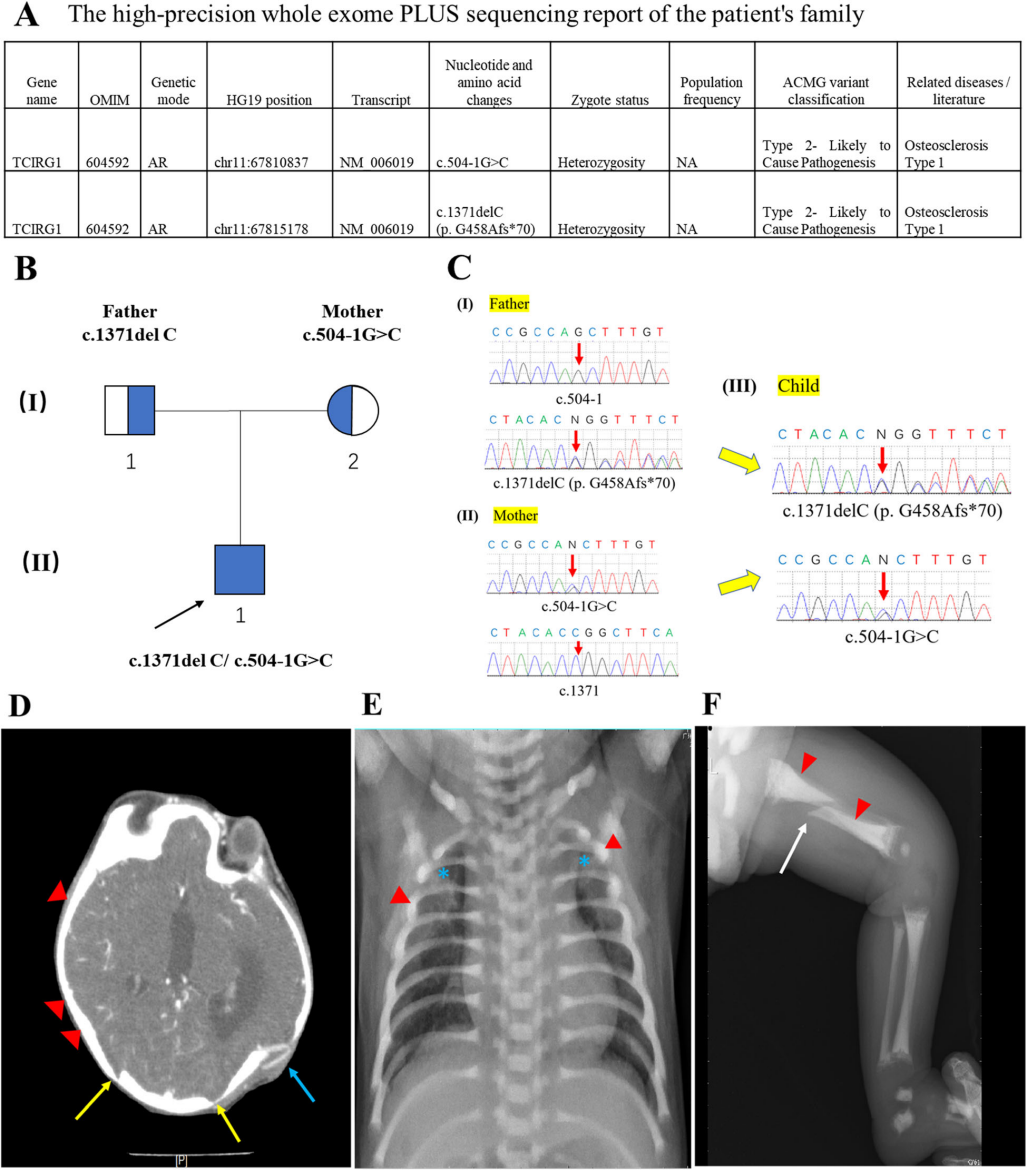
The genetic and clinical characteristics of infantile malignant osteopetrosis patient. **A**. The high-precision whole exome PLUS sequencing report of the patient’s family. **B**. Pedigree of the Chinese family with patient carried c.1371delC/c.504-1G > C compound heterozygous variations in gene TCIRG1. Half-blue symbols represent healthy carriers with a heterozygous mutation. Filled blue symbols refer to patients with autosomal recessive disease. Black arrow indicates the proband. **C**. Sanger sequencing chromatograms from genomic cDNA confirmed the compound heterozygous variations of the patient. **D**. The blue arrow points to the postoperative changes in cranioplasty, the yellow arrow points to the closed cranial suture, and red triangle points to the irregular skull shape and bone sclerosis in the radiograph of head. **E**. Blue stars show the increased lung texture, and red triangles point to the increased bone density in the chest radiograph. **F**. White arrow points the femoral shaft fracture, and increased bone density. Red triangles point to the osteosclerosis of cortical bone in the radiograph of the left leg

The patient with IMO was a 4-month-old male infant, who was admitted to hospital due to a cranial suture closure at the 1st month of his birth. In a prenatal examination at week 24, an abnormal skull shape was found in this child. The child was prematurely born through cesarean section at around 36 weeks’ gestation. He weighed 2490 g at birth and had an abnormal skull development within 35 cm of head circumference. A high-precision whole exome sequencing was ordered for the patient and his biological parents at day 18. The genetic results showed that both parents were normal heterozygous. However, two heterozygous mutation sites of gene TCIRG1 were found in the child. The mutations were c.504-1G > C and c.1371delC (p.G458Afs*70). The c.504-1G > C implied Guanine at the 503 site of intron was mutated to be Cytosine. The c.1371delC (p.G458Afs*70) indicated a deletion of Cytosine at the 1371 site of exon, which caused Glycine to Alanine mutation at position 458, as well as the gene translation termination at position 70 **(**Fig. 1A). These two mutations were inherited from his parents **(**Fig. 2A-C). The head MRI examination was performed at day 50. The results showed that his skull was still irregular in shape (Fig. 2D).

### Laboratory investigations

Clinical characteristics of blood diagnostics and biochemical detection index were shown in Table 1-2.

**Table 1:** Clinical characteristics of blood diagnostics (*n* = 13), median (IQR)

Parameters	Measured values	Normal range
Absolute number of white blood cell, median (IQR), 10^9^/L	18.40 (15.40–21.95)^a^	5–12
Absolute number of lymphocytes, median (IQR), 10^9^/L	10.40 (9.35–12.47)^a^	1.55–4.8
Percentage of lymphocytes, median (IQR), (%)	57.00 (52.00–66.50)^a^	31–40
Percentage of neutrophil, median (IQR), (%)	26.00 (20.00–35.50)^b^	40–60
Absolute number of red blood cell, median (IQR), 10^12^/L	3.54 (3.35–4.42)^b^	4–4.5
Hematocrit, median (IQR), (%)	32.80 (28.80–38.00)^b^	40–50
Hemoglobin, median (IQR), g/l	91.00 (86.50–114.50)^b^	105–145
Mean hemoglobin amount, median (IQR), pg	25.70 (24.90–26.30)^b^	27–34
Mean hemoglobin concentration, median (IQR), g/l	306.00 (282.50–315.00)^b^	316–354
Platelet distribution width, median (IQR), (%)	11.60 (10.80–13.05)^b^	14.8–17.2

The table shows the statistically significant differences between measurement results and normal range (*p* < 0.05). ^a^ The values are higher than normal range. ^b^ The values are lower than normal range

**Table 2:** Clinical characteristics of biochemical detection index (*n* = 4), median (IQR)

Parameters	Measured values	Normal range
Calcium, median (IQR), mmol/L	1.42 (1.27–1.93)^b^	2.24–2.74
Albumin, median (IQR), g/L	36.30 (31.13–44.78)^b^	40–55
α-Amylase, median (IQR), U/L	14.50 (9.75–21.50)^b^	35–135
Globulin, median (IQR), g/L	14.50 (13.20–20.30)^b^	20–40
Creatinine, median (IQR), μmol/L	8.50 (6.50–9.75)^b^	18–97
Total serum protein, median (IQR), g/L	55.00 (45.13–60.08)^b^	65–85
Urine calcium, mmol/ L	0.2^b^	–
25 hydroxyvitamin D, nmol/L	70.20	50–150
Lactic dehydrogenase, median (IQR), U/L	591.50 (425.00–665.00)^a^	159–322
α-Hydroxybutyric dehydrogenase, median (IQR), U/L	497.50 (354.30–583.80)^a^	239–288
Creatine kinase isoenzyme, median (IQR), U/L	50.00 (32.50–66.75)^a^	0–37
Parathyroid hormone, pmol/L	21.3^a^	1.2–7.1
Phosphorus, mmol/L	1.99^a^	1.29–1.94
Total alkaline phosphatase, U/L	534^a^	118–390
Alkaline phosphatase, median (IQR), U/L	448.50 (335.80–775.00)^a^	118–390
Bone alkaline phosphatase, U/L	250^a^	0–200

The table shows the statistically significant differences between measurement results and normal range (*p* < 0.05). ^a^ The values are higher than normal range. ^b^ The values are lower than normal range

### Radiologic results

The result of head radiograph of the patient revealed an irregular skull shape, the closed cranial suture, and the skull bone dense sclerosis, all of which were characteristic patterns of IMO (Fig. 2D). The result of his chest radiograph showed an increased lung texture on both sides of his lung due to the pneumonia, and a general increased density of rib bone (Fig. 2E). Generally, the femur in a 4-month-old child was composed of 36% cortical and 64% trabecular bone [11], whereas the femur of this IMO patient contained at least 50% cortical bone. The radiograph of his left leg showed a complete fracture of femoral shaft (Fig. 2F). Additionally, the bone marrow cavity was narrowed considerably. The result of his femoral radiograph also revealed an abnormal thickening of cortical bone and over mineralization of the trabecular bone, which was presented by a hyper bone density in bone marrow cavity and cortical bone (Fig. 2F).

### Physical examination

This child was 60 cm in height, and 4 kg in weight. His skull was abnormally enlarged. He was fully conscious and had a normal reaction to his surroundings, such as the stimulation of light and sound. His anterior and posterior fontanels were closed, yet the cranial nerve examination was normal.

### Postoperative treatment

The cranioplasty on his left skull was performed smoothly at day 109. Three weeks later, he returned hospital for review and to perform the second cranioplasty on his right skull. However, we found a cerebrospinal fluid leakage in his brain. Therefore, we performed the ventriculoperitoneal shunt surgery on the left side of his head to treat the leakage. Unfortunately, this child had developed a severe pneumonia during his recovery period. And a fracture of his left femoral shaft was happened at month 4. After the treatment of his fracture, the child was discharged.

## Discussion and conclusion

IMO is a rare and heterogeneous genetic disorder characterized by the denser bone mass that is a consequence of defective osteoclast in function and development. In our case, the pathogenic mechanism of the TCIRG1 gene mutation is as follows. The c.504-1G > C mutation occurred in an intron of gene TCIRG1. Although this intron is not involved in the amino acid encoding, this mutation site may have an unknown effect on gene TCIRG1 expression. In addition, the protein V-type proton ATPase subunit an isoform 3 (V-ATPase a3, VPP3, UniProtKB: Q13488) is translated by gene TCIRG1 and contains a transmembrane-helical structure encoded by the amino acid sequence from site 439 to 468 [12]. The c.1371delC (p.G458Afs*70) mutation represents Cytosine base deletion at position 1371 in this gene. This mutation causes the Glycine at site 458 mutates to Alanine. Thereby, the change of this amino acid prevents the formation of transmembrane-helical structure (Black flat line shown in Fig. 1A).

In mammals, V-ATPase consists of at least 13 different subunits, including the cytoplasmic V1 domain and the membrane-embedded V0 domain. The V1 domain is responsible for the hydrolysis of ATPase, and the V0 domain is responsible for a proton transporter [13, 14]. Among the four isozymes of the V0 domain, the V-ATPase a3 is highly expressed in osteoclasts and can promote osteoclasts absorbing bone tissues [15]. This V-ATPase complex located mainly in the osteoblast membrane can promote the function of osteoblast to release protons and generate an acidic environment, which is necessary for hydroxyapatite dissolution in the ruffled border of bone. The mutated TCIRG1 cannot be translated to form the correct V-ATPase complex on the osteoclast membrane, preventing proper protons release that is vital to the formation of an acidic environment **(**Fig. 1B). Therefore, the dysfunction of osteoclast for bone absorption could be an important cause for IMO. This highlights the potential role of these two mutations in gene TCIRG1 in the onset of osteopetrosis.

Here we present the case of a new diagnosis of IMO in a 4-month-old Chinese male infant with the novel splicing site mutation (c.504-1G > C) and the novel frameshift mutation [c.1371delC (p.G458Afs*70)] in TCIRG1. The mutation c.504-1G > C is located in the mRNA splicing region of gene TCIRG1 and the sequence is highly conserved. Although it has never been reported in the gnomAD, other variants at the same position were found. Computational algorithms also predicted that this change may affect splicing function. Our computational work supports our hypothesis that this mutation may affect a splice site in this patient. In addition, the frameshift mutation c.1371delC (p.G458Afs*70) is predicted to be an early termination of protein synthesis. We have found that the p.G458S is a previously reported missense variant associated with osteopetrosis [16–18]. And this variant can be found in gnomAD. However, the mutation c.1371delC (p.G458Afs*70) is not in gnomAD. These reported variants at this position support our hypothesis that the mutation at that residue is associated with osteopetrosis. In summary, based on the clinical manifestations and pedigree analysis of the applicants, according to the ACMG (American College of Medical Genetics) Variation Classification Guidelines, these two mutations are classified to a moderate piece of evidence for pathogenicity (PM2) [19].

In Table 1 we show that the amount of the white blood cells from this child was higher than normal, possibly due to pulmonary inflammation. The decrease in red blood cells and hemoglobin are associated with the physiological anemia or nutritional deficiencies. In Table 2, we show that the hypocalcemia and lower urine calcium are associated with the abnormal calcium consumption caused by osteosclerosis. The higher serum phosphorus and parathyroid hormone maybe associated with the secondary changes of hypocalcemia. Bone metabolism-related indicators, such as the total alkaline phosphatase and bone alkaline phosphatase are measured after fracture and are higher than normal. This can be attributed to the increased bone turnover of fracture healing**.** These symptoms, though atypical, are suggestive of potential osteosclerosis disease. As such, we performed whole exome sequencing on the patient. The whole exome sequencing result validated our speculation and led to the identification of these two novel mutations reported herein. Thus, we propose that to facilitate with the timely diagnosis of IMO, it is beneficial to perform whole exome sequencing in prenatal exams when necessary.

There are several limitations and advantages to our study. First, we report only one IMO patient with two mutations of TCIRG1. To better establish the significance of these mutations in the development of IMO, more cases of this type should be reported in the future. Second, we are unable to track this patient any further due to privacy issues. Thus, the subsequent development of IMO during the life-span of this patient cannot be monitored. Finally, though we firstly reported and conducted primary analysis on the two mutations of TCIRG1: c.504-1G > C and c.1371delC (p.G458Afs*70), the functional and regulatory mechanism of these two mutations should be further studied to fully establish the link between these mutations and the onset of IMO.

To summarize, we report a novel splicing mutation c.504-1G > C and a novel frameshift variant c.1371delC (p.G458Afs*70) in TCIRG1 that have not been previously reported in and outside of Chinese population in gnomAD. The use of whole exome sequencing to detect these two mutations will improve the identification and early diagnosis of IMO, specifically the identification of homozygous individuals with TCIRG1 gene mutation. These mutations in gene TCIRG1 we identified could be a novel therapeutic target for the IMO.

## Data Availability

The datasets used and/or analyzed during the current study are available from the corresponding author on reasonable request.

## Abbreviations

IMO: Infantile malignant osteopetrosis
ARO: Autosomal recessive osteopetrosis
TCIRG1: T-cell immune regulator 1
gnomAD: Genome Aggregation Database
ACMG: American College of Medical Genetics
